# Trial in Elderly with Musculoskeletal Problems due to Underlying Sarcopenia—Faeces to Unravel the Gut and Inflammation Translationally (TEMPUS-FUGIT): protocol of a cross-sequential study to explore the gut-muscle axis in the development and treatment of sarcopenia in community-dwelling older adults

**DOI:** 10.1186/s12877-023-04291-5

**Published:** 2023-09-26

**Authors:** Laurence Lapauw, Jolan Dupont, Nadjia Amini, Laura Vercauteren, Sabine Verschueren, Jos Tournoy, Jeroen Raes, Evelien Gielen

**Affiliations:** 1 Department of Public Health and Primary Care, Division of Gerontology and Geriatrics, KU Leuven, Herestraat 49, Leuven, 3000 Belgium; 2 Department of Geriatric Medicine, UZ Leuven, Leuven, Belgium; 3 Department of Rehabilitation Sciences, KU Leuven, Leuven, Belgium; 4 Department of Microbiology, Immunology and Transplantation, KU Leuven, Leuven, Belgium

**Keywords:** Sarcopenia, Older adults, Community-dwelling, Cross-sectional, Longitudinal, Gut microbiota, Dysbiosis, Intestinal inflammation, Systemic inflammation, Anabolic interventions

## Abstract

**Background:**

Gut microbiota (GM) might play a role in muscle metabolism and physiological processes through a hypothesized gut-muscle axis, influencing muscle mass and function and thus, sarcopenia. The Trial in Elderly with Musculoskeletal Problems due to Underlying Sarcopenia—Faeces to Unravel the Gut and Inflammation Translationally (TEMPUS-FUGIT) aims to explore the gut-muscle axis in sarcopenia.

**Methods:**

First, in a cross-sectional case–control phase, 100 community-dwelling adults without sarcopenia will be compared to 100 community-dwelling adults (≥ 65 years) with sarcopenia of similar age-, gender and BMI-ratio, participating in the ongoing ‘Exercise and Nutrition for Healthy AgeiNg’ (ENHANce; NCT03649698) study. Sarcopenia is diagnosed according to the European Working Group on Sarcopenia in Older People 2 (EWGSOP2) criteria. GM composition and intestinal inflammatory markers (fecal calprotectin, lactoferrin and S100A12) will be determined in fecal samples. Systemic inflammatory markers (hs-CRP, IL-4, IL-6, TNF-α, IL-13, IL-1β and creatine kinase) will be determined in fasted blood samples. Both groups will be compared using appropriate statistical testing, whereas linear regression will be used for cross-sectional associations between gut, inflammatory and sarcopenia parameters.

Second, in the longitudinal phase, sarcopenic older adults will be requested to deliver five fecal samples during the 12-week intervention to assess the effects of protein, omega-3 and a physical exercise program on the GM.

**Discussion:**

TEMPUS-FUGIT aims to explore the gut-muscle axis by comparing GM composition between sarcopenic and non-sarcopenic older adults and to determine the association of GM with intestinal and systemic inflammatory markers and sarcopenia-defining parameters (muscle mass, muscle strength and physical performance). Furthermore, effects of single or combined, optimized and individualized anabolic interventions (exercise, protein and omega-3 supplementation), on GM will be explored in persons with sarcopenia.

TEMPUS-FUGIT aims to impact clinical practice by clarifying the relationship between the gut-muscle axis and sarcopenia. TEMPUS-FUGIT is expected to contribute to the discovery of clinical and microbial biomarkers for sarcopenia and insights in its pathophysiology, opening possible future perspectives for novel sarcopenia treatment strategies targeting GM.

**Trial registration:**

ClinicalTrails.gov NCT05008770, registered on August 17, 2021; first participant enrolled on September 21 2021.

**Supplementary Information:**

The online version contains supplementary material available at 10.1186/s12877-023-04291-5.

## Background

Sarcopenia is a progressive skeletal muscle disorder characterized by the age-related loss of muscle mass and function, leading to ‘muscle failure’. When no other factors than aging trigger the muscle loss, sarcopenia is considered ‘primary’ [1–4]. Sarcopenia is highly prevalent and predisposes older adults to deleterious health outcomes such as disability, mobility limitations, fractures and mortality [5–9].

Previous research has linked altered gut microbiota (GM) with inflammatory and age-related diseases such as Alzheimer’s disease and Morbus Parkinson [10]. GM are an overall resilient but complex ecosystem of viruses, fungi, parasites, yeasts and bacteria, symbiotically living in the human intestine, reaching peak diversity and richness in the colon [11–13]. The composition of the GM rapidly changes before the age of 3 years, stabilizes in adulthood and alters with aging [14]. Healthy GM regulate physiological processes through several mediators i.e. Short Chain Fatty Acids (SCFA), antioxidants and cytokines [12]. Normal aging is associated with decreased biodiversity and overgrowth of pathobionts, defined as ‘dysbiosis’, leading to a decrease of the aforementioned mediators [15].

Recent studies suggest that GM also play a role in muscle metabolism and physiology through a hypothesized ‘gut-muscle axis’, directly influencing muscle mass and function and hence, sarcopenia. However, some gaps in the literature regarding this gut-muscle axis remain and urge further investigation.

First, prior studies generally reported decreased GM diversity in sarcopenic persons and older adults with low muscle mass compared to respectively non-sarcopenic persons and older adults with normal muscle mass [16–18]. However, inconsistent associations were reported between GM and sarcopenia-defining parameters (muscle mass, muscle strength and physical performance); in addition, various sarcopenia definitions were used in studies that investigated the GM composition of persons with sarcopenia.

Second, disturbed GM might influence the age-related chronic low-grade systemic inflammation or ‘inflammaging’ in older persons, a major driving mechanism behind sarcopenia [19, 20]. Dysbiosis also results in chronic intestinal inflammation. However, data about intestinal inflammation as well as data on the association between GM and both systemic and intestinal inflammation in persons with primary sarcopenia are lacking [21]. In patients with Inflammatory Bowel Disease (IBD), in whom the incidence of sarcopenia is 42%, sarcopenia is associated with altered GM and increased levels of the gut inflammatory markers fecal calprotectin, lactoferrin and S100A12 [22–25]. However, similar data in older adults with primary sarcopenia are lacking.

Third, whereas GM might influence the pathophysiology of sarcopenia, recommended treatments for sarcopenia, i.e., protein supplementation and physical exercise, may, in turn, alter the GM. However, not all data are consistent with a beneficial effect of physical exercise and a high-protein diet on GM. To illustrate, protein supplemented athletes expressed decreased health-related GM, but their levels of health-related mediators, such as SCFA were unchanged [26]. Also, young adults acutely exposed to high levels of physical training expressed increased opportunistic gut pathogens (e.g., Streptococcus) and a pro-inflammatory profile [27, 28]. Nonetheless, results are conflicting, and no data are available regarding the effect of sarcopenia treatment on GM composition in older adults with primary sarcopenia.

**Fig. 1:**
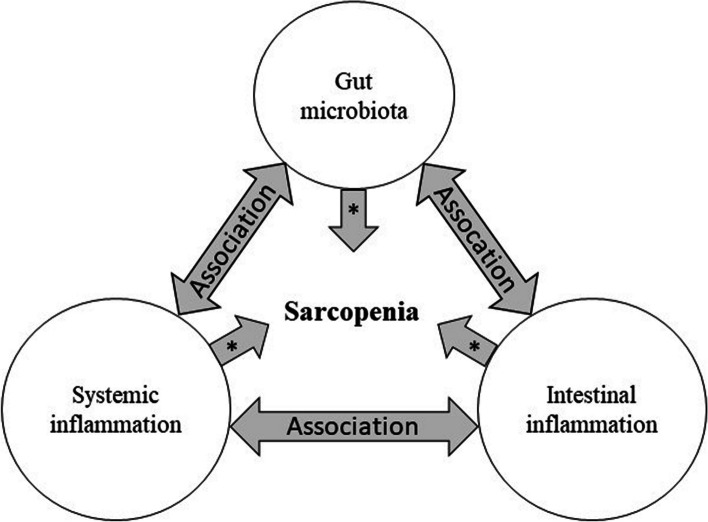
TEMPUS-FUGIT: Exploring the gut-muscle axis in sarcopenia. * Gut microbiota, intestinal and systemic inflammation may contribute to development of sarcopenia

The Trial in Elderly with Musculoskeletal Problems due to Underlying Sarcopenia-Faeces to Unravel the Gut and Inflammation Translationally (TEMPUS-FUGIT) aims to unravel the gut-muscle axis in age-related sarcopenia by addressing these gaps. First, TEMPUS-FUGIT will compare GM composition and intestinal inflammation between a well-defined sarcopenic community-dwelling older population and non-sarcopenic controls. Second, in sarcopenic individuals, associations between sarcopenia-defining parameters and GM, systemic and intestinal inflammation, that all may contribute to sarcopenia, will be explored. An overview of the associations that will be investigated is given in Fig. 1. Finally, TEMPUS-FUGIT will explore the effects of optimized and individualized anabolic treatments for sarcopenia on the GM composition and intestinal inflammation in older persons with sarcopenia. We hypothesize a significant effect of these anabolic interventions on the GM composition in sarcopenic persons.

## Methods/design

### General design

The TEMPUS-FUGIT-project is a single center study conducted in the University Hospitals (UZ) Leuven, Leuven, Belgium. The study set-up is two-fold, consisting of an observational, cross-sectional, case–control phase and a longitudinal, interventional, phase.

In the cross-sectional study phase, the primary objective is to compare GM composition and intestinal inflammatory markers between 100 healthy community-dwelling older adults without sarcopenia and 100 community-dwelling older adults with sarcopenia.

The longitudinal phase of TEMPUS-FUGIT has a triple-blinded randomized controlled trial (RCT) set-up. This comprises a 12-weeks program of single or combined, optimized, and individualized anabolic interventions (a physical exercise program, protein and/or omega-3 polyunsaturated fatty acids (PUFA) supplementation). The effects of these interventions on GM composition and fecal intestinal inflammatory markers will be assessed in 100 sarcopenic older adults. To this end, participants will be 1:1 allocated into one of five intervention groups, which differ in the combination of the exercise and nutritional interventions.

**Fig. 2:**
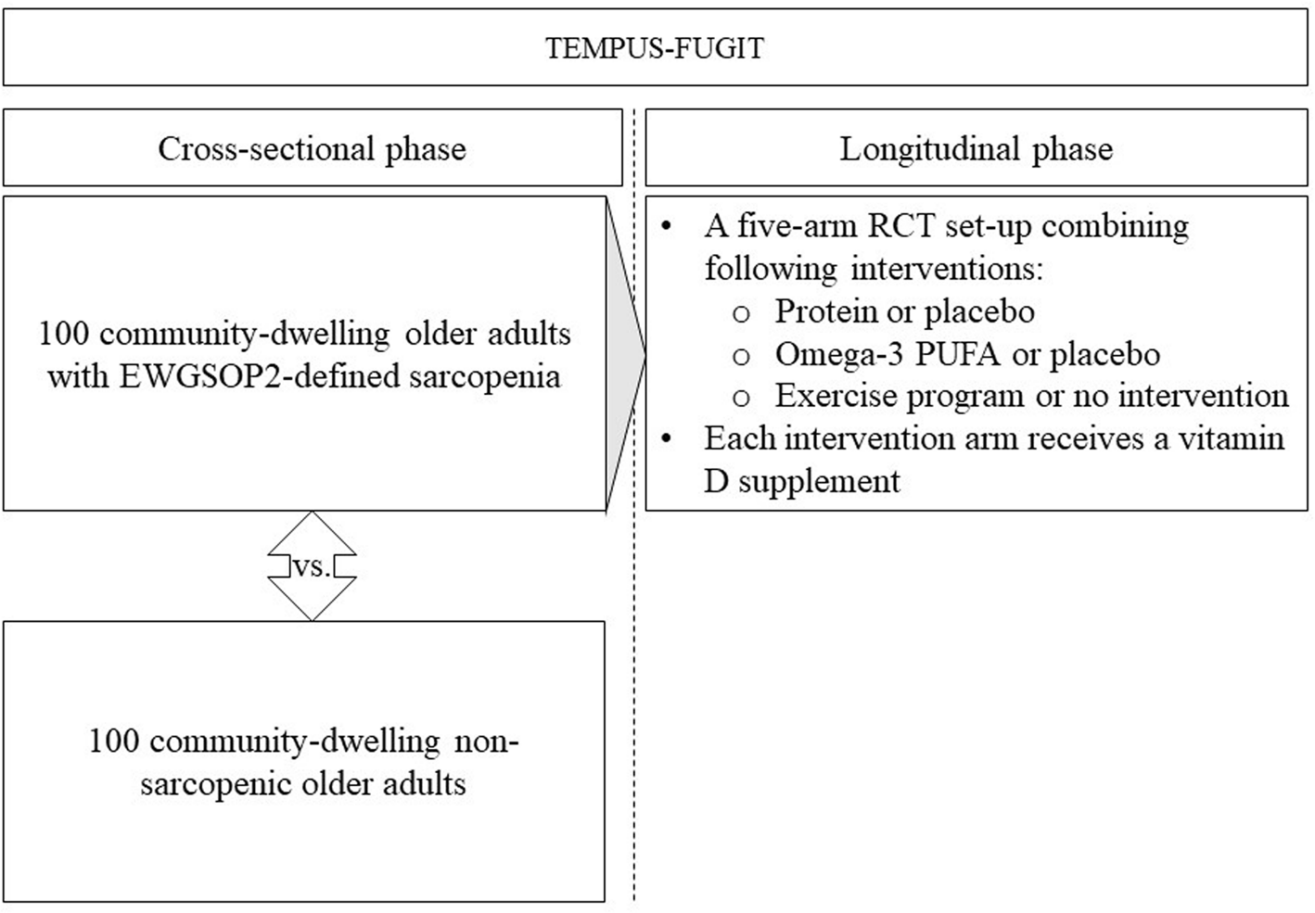
TEMPUS-FUGT general study design PUFA: Polyunsaturated Fatty Acid, EWGSOP2: European Working Group on Sarcopenia in Older People 2

An overview of the general set-up of the TEMPUS-FUGIT study is given in Fig. 2. More details on the interventions have been previously published by Dedeyne et al. and will be summarized further in this manuscript [29].

### Participants

In TEMPUS-FUGIT 100 healthy community-dwelling non-sarcopenic older adults (aged ≥ 65 years) will be recruited to be compared with 100 older adults with sarcopenia defined according to the revised definition of the European Working Group on Sarcopenia in Older People (EWGSOP2) [30]. The sarcopenic older adults will also participate in the longitudinal (interventional) phase of the study.

The sarcopenic and the non-sarcopenic group will be gender-matched, whereas for age and Body Mass Index (BMI), caliper matching (maximum tolerated difference between matched persons) will be applied [31]. Sarcopenic older adults will be recruited from the ongoing triple blinded RCT ‘Exercise and Nutrition for Healthy AgeiNg’ (ENHANce) study (Clinical Trial number NCT 03649698) [29]. Participants will be recruited from the environment of Leuven, Flanders, Belgium. To ensure recruitment of a sufficient number of study persons, contacts have been established with the geriatric outpatient clinic (including falls prevention clinic) and representatives of service flats and day care centers or organizations for older adults. Furthermore, medical staff collaborating to the project will use their network for recruitment. Also health care specialists, i.e., general practitioners, pharmacists and physiotherapists will be requested to refer patients who may be eligible for this study. Also information about the study will be distributed by organizations and websites that target older persons, advertisements in local newspapers and distributions of flyers. Older adults, who were initially screened for the ENHANce study but failed to meet the inclusion criteria for sarcopenia, will be re-contacted to participate in the non-sarcopenic control group of the TEMPUS-FUGIT study.

### Assessment of eligibility criteria

Sarcopenic participants should be diagnosed with probable (low muscle strength), confirmed (low muscle strength & mass) or severe sarcopenia (low muscle strength, mass and physical performance) by EWGSOP2 [30], whereas healthy controls should not have sarcopenia. Furthermore, controls as well as sarcopenic participants should meet the following inclusion criteria: ≥ 65 years, community-dwelling or assisted living, able to communicate in Dutch both orally and written, have a Mini-Mental State Examination (MMSE) Score ≥ 21 points, be in the possession of a freezer for preservation of fecal samples, absence of diabetes and no intake of diabetes medication, no intake of corticoids, absence of terminal illness with prognosis < 6 months, no antibiotics use in the past 3 months, absence of ongoing or previous (< 5 years) malignancy of the gastrointestinal tract, no Irritable Bowel Syndrome (IBS) or IBD, no chronic kidney disease (glomerular filtration rate (eGFR) < 30 ml/min/1.73m^2^) and absence of disorders, recent complaints, injury or surgery that prevent participation to the study according to the researchers. Additionally, sarcopenic participants should not have followed a rehabilitation or physical exercise program > 2/week in the last six months and should have a protein intake ≤ 1.5 g per kg body weight (BW) per day.

### Study procedures

#### Screening visit

TEMPUS-FUGIT starts with a screening visit, where study procedures will be explained, eligibility criteria will be checked and informed consent will be obtained by one of the researchers. If the participant qualifies for the study, weight (SECA, model no 8801321009, SECA UK Ltd.) will be measured to the nearest 0.1 kg and height (Harpenden stadiometer Holtain Ltd., Crosswell UK) to the nearest 0.1 cm, measured barefoot, standing upright with clothes on. BMI is calculated through weight/height^2^ (kg/m^2^). Hand grip strength (HGS) will be measured with a handheld dynamometer (Jamar 1, TEC Inc., Clifton, NJ, USA) while the participant is seated, holding the arm in a 90° angle. According to the Southampton protocol, HGS is measured six times for alternating hands and the maximal value is reported [32, 33]. The chair stand test (CST) determines leg muscle strength by measuring the time it takes for the participant to rise five successive times out of a chair as fast as possible without using any support and holding the arms crossed in front of the chest. Cut-off values for low muscle strength according to EWGSOP2 (and, hence, probable sarcopenia) are > 15 s for the CST for both sexes and a HGS < 27 kg and < 16 kg for men and women respectively [5]. Finally, when no recent lab results (12 weeks old) are available, an additional fasted sample will be taken in order to check whether the participants fasted glucose levels are < 126 mg/dl or HbA1C < 6.5%, to exclude diabetes mellitus [34].

#### Preparation period

When the participant is diagnosed with at least probable sarcopenia based on the EWGSOP2-defined low muscle strength and meets the inclusion criteria, the participant is eligible for both the cross-sectional phase and the longitudinal phase of TEMPUS-FUGIT.

For the longitudinal phase, the sarcopenic participants will be 1:1 block randomized into one of the five intervention groups, as previously described by Dedeyne et al. [29]. All these participants will be requested to take 800 International Units (IU) of vitamin D and one capsule of omega-3 PUFA/placebo per day, starting one month before the baseline test visit. Participants will also be asked to keep a food diary during four non-consecutive days in order to individually supplement protein intake until the recommended daily allowance (RDA) for older adults with chronic conditions (i.e., 1.5 g/kg BW/day) [35]. Furthermore, these food diaries will be analyzed to obtain the participants’ habitual protein and energy intake, and to assess possible associations with GM composition. Five days prior to the baseline test visit, participants will be requested to start to take their individualized protein supplements or placebo powder and will wear an inertial measurement unit (IMU) (MoveMonitor + , McRoberts, The Netherlands) to monitor physical activity. Participants will be requested to register the intake of the vitamin D, omega-3/placebo and protein/placebo supplement in a trial diary during the intervention. In the week prior to the baseline test visit, participants need to collect two fecal samples produced at two separate moments with the provided material (Laboratory of Molecular Bacteriology, KU Leuven/Rega Instituut, Vlaams Instituut voor Biotechnologie (VIB), Belgium). Immediately after sample collection, the participant fills out the Bristol Stool Score Chart [36] to determine fecal consistency and preserves the samples at -20°C [37]. Finally, participants will be requested to complete a general health questionnaire as well as the ROME IV questionnaire to screen for IBS symptoms [38].

When the participant does not meet the EWGSOP2-criteria for (probable) sarcopenia, the participant will be included as a non-sarcopenic control person in the cross-sectional phase of the TEMPUS-FUGIT study. Non-sarcopenic participants will follow the same preparation period as described above, being requested to complete food diaries and health questionnaires, to collect two stool samples and wear the IMU, but without being block randomized to the interventions (nutritional supplementations and/or the exercise program).

#### Baseline test visit

For both the non-sarcopenic controls and the sarcopenic participants, a test visit at the study center will follow the preparation period. During this visit, the four-day food diary, the IMU and the two frozen fecal samples brought along by the participant (avoiding thawing using ice packs) will be collected and fasted blood samples will be taken.

- The fecal samples will immediately be stored at -80 °C until analysis of intestinal inflammatory markers (fecal calprotectin, lactoferrin and S100A12) and GM composition. Quantitative Microbiome Profiles (QMP) will be obtained through the following extensive -omics analysis. Targeted (16S/18S) amplicon sequencing analysis of the V3-V4 variable region of the 16S rRNA gene, via reverse polymerase chain reaction (RT-qPCR) technique will provide information about GM taxonomic composition, i.e., the type of GM present, and their abundance.. Metagenomic analysis (comprehensive assessment of DNA of the complete content of a clinical sample) will be performed through whole genome shotgun analysis using next generation platforms. Intestinal inflammatory markers will be determined through Enzyme Linked Immunosorbent Assay (ELISA).
- In fasted blood samples, the following parameters will be determined: systemic inflammatory parameters (hs-CRP, IL-4, IL-6, IL-13, IL-1β, TNF-α and creatine kinase), muscle markers (myostatin, activin A, irisin, apelin), clinical parameters (hemoglobin, eGFR, serum albumin, fasting glucose, red blood cell count, hematocrit, Mean Corpuscular Volume (MCV), Mean Corpuscular Hemoglobin (MCH), Mean Corpuscular Hemoglobin Concentration (MCHC), white blood cell count and formula, platelet count, hemoglobin A1c (HbA1c), ferritin, aspartate aminotransferase (AST), alanin transaminase (ALT), γ-glutamyltransferase, total and direct bilirubin, total protein, serum protein electrophoresis, uric acid, sedimentation rate, triglycerides and total, high-density lipoprotein (HDL) and low-density lipoprotein (LDL) cholesterol, and hormonal status (25-hydroxyvitamin D, vitamin B12, morning cortisol, insulin, insulin-like growth factor (IGF-1)).

Clinical parameters measured during this visit are the participants’ appendicular lean mass (ALM) by Dual X-Ray-Absorptiometry (DXA) (Horizon A scanner, Hologic Inc., Bedford, MA, USA) in a fasted state. Also, bioelectrical Impedance Analysis (BIA) will be used to assess body composition (Tanita TBF-300, Tanita Coorporation, Tokyo Japan; Omron BF-300, Omron Healthcare Co, Ltd., Kyoto Japan; BodyStat 1500, Bodystat, Isle of Man, UK; Bodystat Quadscan 4000, Bodystat, Isle of Man, UK) in a fasted state. Hand grip strength will be assessed with a hand dynamometer as described above. In the upper dominant leg, flexor and extensor muscle strength will be assessed with isometric (60° and 90°), isotonic (40,20 1 and 60% of individual 1- repetition maximum (1-RM) and isokinetic (60°/s and 180°/s) BIODEX (Biodex system 3 Pro Multijoint System) measurements as previously described by Baggen et al. [39]. Two minutes of rest will be provided between these three BIODEX measurements. Axillary body temperature (Predictor, GEON Corporation, Chang Hwa Hsien, Taiwan) and blood pressure will be determined (Microlife, Microlife AG Swiss Corporation, Widnau Switzerland). Physical performance and balance will be assessed with the Short Physical Performance Battery (SPPB) [40], the Mini-Balance Evaluation System Test (Mini-BESTest) and the gait speed test [41]. Gait speed will be determined over a six-meter distance, only timing the inner four meters. Participants will be requested to walk at their usual pace. Low gait speed is defined at the cut-off of ≤ 0.8 m/s. Furthermore, the physical frailty stage (Fried criteria), activities of daily living (ADL; Barthel index) [42], quality of life (QoL; two questionnaires: 36-Item Short Form Health Questionnaire (SF-36) [43] and SarQoL [44]), number of falls, fall circumstances and fear of falling (Falls Efficacy Scale-International (FES-I) questionnaire [45]) will be evaluated. Cognitive performance will be assessed with the Repeatable Battery for Assessment of Neurological Status (RBANS) (measuring language, visuospatial skills, attention and immediate and delayed memory) [46], the Trail-Making-Test (TMT) [47], the Stroop test and Maze-test [48]. Finally, nutritional status will be assessed with the Mini-Nutritional Assessment-Short Form (MNA-SF) [49]. Participants who successfully complete the study test visit will be offered a gift voucher worth 15 euros.

#### Intervention period

**Fig. 3:**
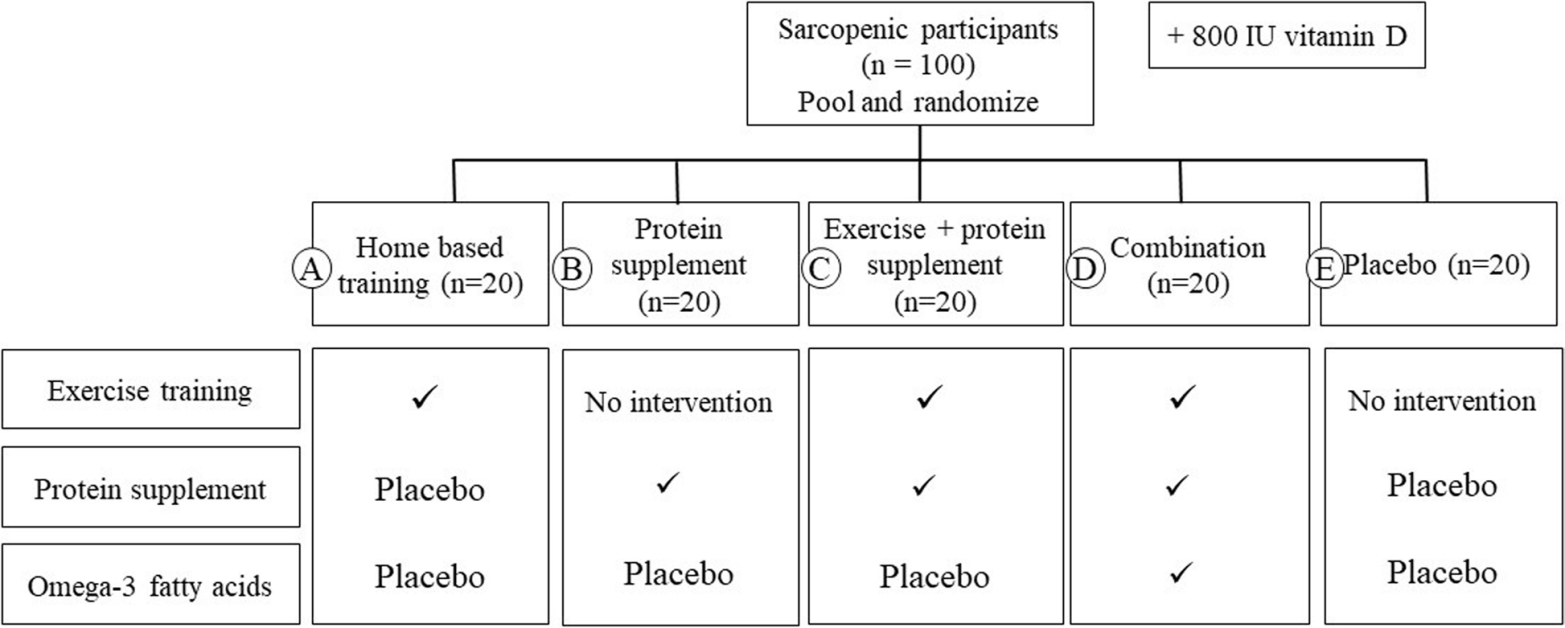
Overview on the anabolic interventions in the longitudinal phase of TEMPUS-FUGIT. Each group will receive 800 International Units (IU) of vitamin D

In the longitudinal RCT phase of the TEMPUS-FUGIT study, sarcopenic older adults will be block randomized to one of five interventional programs, stratified according to sex, via a sealed opaque envelope by an independent researcher. Participants in each of the interventional groups will follow a 12-week intervention period as previously described [29]. Briefly, the interventions consist of a training program and/or nutritional supplementation. The exercise intervention comprises an adapted version of the home-based Otago exercise program (OEP) consisting of warming-up, balance, strength and stretching exercises together with a walking program. The OEP has been proven to effectively reduce falls in older community-dwelling adults. Currently the OEP is being investigated in the context of sarcopenia, reporting a preliminary beneficial effect [50, 51]. The protein intervention consists of a personalized amount of protein supplementation, rich in leucine (Resource instant protein, Nestlé), aiming to reach the RDA of 1.5 g/ kg BW/day, spread equally over the day [35]. The placebo protein is isocaloric digestible maltodextrin (Resource dextrin maltose, Nestlé). Omega-3 PUFA supplements consist of 450 mg docosahexanoic acid (DHA) and 500 mg of eicosapentanoic acid (EPA) per day. The omega-3 PUFA placebo contains 1 g peanut oil. All groups receive vitamin D supplementation (800 IU cholecalciferol). Researchers and participants will be blinded to the nutritional supplements, but not to the exercise program. The statistician is blinded to all the interventions. At each contact moment during the intervention, researchers encourage participants to comply to the interventions. An overview of the study design/ intervention groups is given in Fig. 3.

In addition to the two fecal samples delivered during the preparation period, sarcopenic participants will be requested to deliver an additional fecal sample and to complete a medical and the ROME IV questionnaire at week 4, week 8 and week 12 of the intervention period. At weeks 1, 4 and 8 additional non-fasted blood samples will be taken to assess clinical parameters. Also axillary body temperature, blood pressure, length, height and waist and hip circumference will be determined at these timepoints. At week 12, muscle mass, strength, physical performance, QoL, nutritional status and cognitive performance will be assessed using the same testing program as performed during the baseline visit. A complete overview of all the study procedures and a timetable is given in Table 1.

**Table 1:** Study procedures and timeline

Study week (W)	All participants			Sarcopenic participants						
	Screening	Preparation	Baseline	Intervention period						
				W1	W2	W4	W6	W8	W10	W12
Fecal sample, MQ^m^ and ROME IV^n^ questionnaire		✓				✓		✓		✓
Eligibility criteria, informed consent, alcohol intake, smoking, educational and marital status	✓									
Muscle strength (Biodex), muscle mass^a^ (DXA, BIA), SPPB^b^, BMI^c^			✓							✓
Physical activity by IMU^d^		✓	✓	✓	✓	✓	✓	✓	✓	✓
Exercise compliance^d,e^ and MiniBesTest^d,e^			✓	✓	✓	✓	✓	✓	✓	✓
SF-36^f^ and SarQol^g^, ADL^h^, frailty^i^, Short-FES-I,^j^ cognitive test battery^k^			✓							✓
Food diary analyses to assess associations between GM and habitual protein intake		✓		✓		✓				✓
Falls, health care use			✓	✓	✓	✓	✓	✓	✓	✓
MNA-SF^l^			✓				✓			✓
Body temperature, waist & hip circumference, blood pressure, weight, length			✓			✓		✓		✓
Blood sample	✓		✓	✓		✓		✓		✓

^a^:assess muscle mass with Dual X-ray Absorptiometry and Bio-electrical Impedance Analysis

^b^:Short Physical Performance Battery

^c^:Body Mass Index

^d^:assess physical activity level by movement monitor

^e^:only participants in the exercise group

^f^:SF-36 health related quality of life scale questionnaire

^g^:SarQol questionnaire

^h^:Barthel index to assess Activities of Daily Living (ADL)

^i^:frailty defined according the Fried criteria

^j^:Short Falls Efficacy Scale international

^k^: cognitive tests comprising: Repeatable Battery for the Assessment of Neuropsychological Status (RBANS), Trail-Making-Test (TMT) part A and B, Maze test, Stroop test

^l^:Mini nutritional assessment short form

^m^: general medical questionnaire

^n^: questionnaire to screen for Irritable Bowel Syndrome (IBS)

### Outcome measurements

For the cross-sectional phase, outcome measurements will be taken at the baseline test visit.

- The primary outcomes in this phase are:
  - Differences in multiple GM α-, and β-diversity indices, enterotypes, bacterial cell counts and abundance of specific taxa between sarcopenic older adults and non-sarcopenic controls using two fecal samples collected in each group at the baseline test visit [52, 53]. Both GM diversity indices and measures for expression of bacterial abundance will be specified in the section “Assessment of GM composition and statistical analyses” further below.
  - Differences in the levels of the intestinal inflammatory markers fecal calprotectin, lactoferrin and S100A12 between sarcopenic older adults and non-sarcopenic controls using two fecal samples collected in each group at the baseline test visit.
- Secondary outcomes assessed in sarcopenic older adults are:
  - Associations between GM composition and clinical parameters: 1) ALM (by whole body DXA and BIA), muscle strength (HGS, CST and knee strength) and physical performance (gait speed and SPPB); 2) protein intake by four-day food diaries and risk of malnutrition by the MNA-SF.
  - Associations between GM and levels of systemic (hs-CRP; IL4, IL6, IL-13, IL-1β, TNF-α, and creatin kinase) and intestinal (fecal calprotectin, lactoferrin and S100A12) inflammatory markers.
  - Associations between levels of systemic and intestinal inflammatory markers.
  - Associations between fecal calprotectin, lactoferrin, S100 and respectively: 1) aforementioned systemic inflammatory markers; 2) ALM (whole-body DXA), muscle strength (HGS, CST, knee strength) and physical performance (gait speed, SPPB); 3) systemic muscle biomarkers (myostatin, activin A, irisin, apelin).

For the longitudinal phase in sarcopenic participants, outcome measurements will be collected from the baseline test visit until the week 12 visit. The primary outcome of this phase is:

- Change in GM composition between the protein intervention group (group B, Fig. 3) and the placebo group (group E).

### Secondary outcomes:

- Change in intestinal inflammatory markers between the placebo and protein supplement group (group E vs group B).
- Changes in GM composition and intestinal inflammatory markers between other treatment groups (group A vs group E; group C vs group E; group D vs group E).
- Associations between baseline GM composition and change in intestinal inflammation or sarcopenia-defining parameters.

### Assessment of GM composition and statistical analyses

GM analyses will comprise determination of within sample diversity (α-diversity), including sample richness, taxa abundance, evenness, and taxa distribution through the following indices: Chao 1, Shannon and Simpson’s indices, abundance-based coverage estimators (ACE) index and the Good’s Coverage index. Taxa abundance will be expressed as Operational Taxonomic Units (OTU) and/or Amplicon Sequence Variants (ASV). Sequencing reads will be demultiplexed with LotuS (version 1.565) and processed further with the DADA2 pipeline (version 1.6.0), using the RDP classifier version 2.12 for taxonomy assignment (default parameters). Between sample dissimilarity (β-diversity) will be determined with Bray–Curtis dissimilarity (through a distance based non-parametrical permutational analysis of variance (PERMANOVA) test) and weighted and unweighted UniFrac (through principal coordinates analysis (PCoA)), respectively. Comparisons of relative GM abundance between sarcopenic and non-sarcopenic older adults will be performed through linear discriminant analysis (LDA) effect size (LEfSe). GM analyses, as well as visualization of the results will be performed using R vegan and phyloseq packages [54]. Enterotyping (or community-typing) will be performed based on the Dirichlet-multi-nominal Model (DMM) approach in R (dmn function) as previously described on a combined genus-level abundance matric containing the study samples as well as 1106 Flemish Gut Flora Project (FGFP) samples to increase accuracy [55, 56]. Continuous data will be checked for normality with the Shapiro–Wilk test. The t-test will be performed for between-group analysis of continuous clinical parameters, if normally distributed. For GM analyses, non-parametric testing will be applied. The Mann–Whitney U test will be performed in case of non-parametric distribution. Correlations between microbiota species and clinical parameters will be assessed with Spearman correlation. Linear regression models adjusted for putative confounders (e.g., age) will be used to check for associations between GM species abundance and clinical parameters. To determine possible effects of the interventions on GM in different groups, linear mixed models will be applied. Multiple testing corrections will be applied if it is applicable (Benjamini-Hochberg- False Discovery Rate (FDR)). An FDR of ≤ 0.10 will be taken into account for GM analyses [57]. Level of significance will be a p-value of ≤ 0.05. A bioinformatician from VIB and a statistician from LBiostat will be consulted for the statistical analyses.

### Sample size

In TEMPUS-FUGIT, a pragmatic sample of 100 sarcopenic people from the longitudinal phase, will be compared with 100 non-sarcopenic volunteers. Therefore, 110 non-sarcopenic controls will be recruited, in order to ensure 100 included control persons that, similarly to the sarcopenic participants, have a protein intake < 1.5 g/kg BW/day. Indeed, based on previous similar data, we expect, a drop-out of 5.88% in the non-sarcopenic control group due to a protein intake > 1.5 g/kg BW/day, [58]. Due to the exploratory character of this study, no sample size calculation was performed. Based on previous studies, 100 participants in each group is considered as sufficient for this study [16, 18, 29].

### Protocol amendment

The first version of the protocol dates from July 7^th^ 2021 and was accepted was to the Ethics Committee of UZ/KU Leuven on July 14^th^ 2021. Modifications to the protocol possibly impacting the study conduct, patient benefits and/or patient safety, changes of study aims, changes of study design, sample size, patient population and study procedures will require an amendment. Any amendment will be approved by the local Ethics Committee (EC) and will be agreed upon by the study team.

### Ethics and dissemination

TEMPUS-FUGIT was approved by the Ethical Committee (S65127) and registered at Clinical Trials.gov (NCT05008770). The researchers obtain written informed consent from each individual willing to participate in the study, according to good clinical practice. With this study protocol, a thorough overview is given of the methodology of the TEMPUS-FUGIT project, based on the Standard Protocol Items, Recommendations for Interventional Trails (SPIRIT) guidelines [59].

Dissemination of the results will take place through publications in scientific and professional journals and by international conferences. Adverse events will be registered by the study coordinator. The study coordinator monitors all data and reports to the principal investigator. All study-related data will be handled and stored confidentially under supervision of the study coordinator. Protocol amendments will be reported on ClinicalTrails.gov. The study is performed an accordance with the principles of the Declaration of Helsinki.

## Discussion

TEMPUS-FUGIT aims to unravel the gut-muscle axis in sarcopenia by a two-fold set-up. First, cross-sectionally, by comparing GM composition and intestinal inflammation between sarcopenic and non-sarcopenic older adults. Second, in a longitudinal RCT set-up, by assessing the effects of combined, optimized, and individualized anabolic treatments for sarcopenia on GM and intestinal inflammation in sarcopenic older adults.

A first strength of TEMPUS-FUGIT is its well-considered design, defining sarcopenia according to the most recent criteria of the EWGSOP 2 [30]. Moreover, this study excludes participants with comorbidities influencing GM (e.g., malignancy, IBS, IBD or chronic kidney disease) and medications influencing muscle (e.g. corticoids or diabetes medications), resulting in an ideal population to explore the gut-muscle axis. Furthermore, this study aims to match both groups according to gender, age and BMI, since these factors influence both GM composition and sarcopenia [18, 60]. Thus, TEMPUS-FUGIT avoids bias related to these parameters, which was not always the case in previous studies investigating muscle mass, strength and GM in older adults [61–63].

Secondly, state-of the art metagenomic sequencing methods (e.g. Quantitative Microbiome Profiling, Shotgun whole genome sequencing) will be used to explore quantitative signals and bacterial functionality, which has not yet been done in an EWGSOP2-defined sarcopenic population [17].

Third, rather than solely comparing GM between non-sarcopenic and sarcopenic older individuals, TEMPUS-FUGIT pioneers in the assessment of intestinal inflammation markers, i.e., S100A12, fecal lactoferrin and fecal calprotectin, with the latter marker having a high specificity and sensitivity for gut inflammation [64]. This study will also investigate the possible associations between intestinal inflammation and respectively systemic inflammation and GM composition and its contribution to the development of sarcopenia, which has, to our knowledge, not yet been investigated.

Fourth, in the longitudinal phase, the effect of single and combined, optimized, and individualized anabolic interventions on GM and intestinal inflammation will be assessed in a well-defined sarcopenic population. To the best of our knowledge, this has not yet been done in community-dwelling older adults with sarcopenia. Optimization and individualization of interventions were based on the state-of the art literature and the RCT design allows to assess causality. Also, dietary intake will be registered through detailed food diaries. Physical activity will be reported in movement diaries as well as objectively measured with the IMU, allowing to account for incompliance to the interventions [16, 64].

Finally, the protein placebo compound administered in this study phase, is digestible maltodextrin (Resource Instant protein, Nestlé). This form of maltodextrin is completely digested before reaching the colon, therefore, not fermenting colon bacteria and not exerting a prebiotic effect, thus avoiding bias and making it a suitable placebo [65]. Unlike this study, previous studies using maltodextrin placebo have insufficiently clarified whether the digestible form is used or not [66].

Some limitations need to be taken into account. First, due to the exploratory character of this study, no power calculations for sample size were conducted. However, previous cross-sectional and case–control studies with sample sizes varying between 17–60 participants per group, reported significant results investigating the GM and muscle in older or sarcopenic adults [18, 67]. Therefore, in TEMPUS-FUGIT, the preset 100 participants in each group should be sufficient to detect significant differences in GM between both groups [16, 18, 68].

Second, due to the single center set-up, TEMPUS-FUGIT might be prone to selection bias. It has been shown that GM composition is influenced by geographic location, population demographics, ethnicity and the ‘exposome’, defined as “the measure of all the exposures of an individual from all sources, including environmental and occupational sources, in a lifetime and how those exposures relate to health” [64, 69, 70]. Hence, caution should be urged to extrapolate findings to the general population.

In conclusion, this study pioneers in its two-fold set-up aiming to further unravel the gut-muscle axis in sarcopenia. This study may open perspectives for research with microbial compounds such as pre-, pro- and symbiotics, targeting a sarcopenia-associated microbial landscape, as an additional treatment option for sarcopenia. We also expect this study to contribute to the discovery of new biomarkers, both clinical and microbial, for sarcopenia, which will help to identify persons at risk of this progressive muscle disease.

### Supplementary Information

**Additional file 1.**

## Data Availability

Not applicable.

## Abbreviations

ACE: Abundance-based Coverage Estimators
ADL: Activities of Daily Living;
ALM: Appendicular Lean Mass;
ALT: Alanin Transaminase
AST: Aspartate aminotransferase
ASV: Amplicon Sequence Variants
BIA: Bioelectrical Impedance Analysis
BMI: Body Mass Index
CST: Chair Stand Test
DHA: Docosahexaenoic Acid
DXA: Dual X-Ray Absorptiometry;
ELISA: Enzyme-Linked Immunosorbent Assay
ENHANce: Exercise and Nutrition for Healthy AgeiNg
EPA: Eicosapentaenoic Acid
EWGSOP2: European Working Group on Sarcopenia in Older People 2
FDR: False Discovery Rate
eGRF: Estimated Glomerular Filtration Rate
GM: Gut Microbiota
HbA1c: Hemoglobin A1c
HGS: Hand Grip Strength
hs-CRP: High sensitivity C-Reactive Protein
GM: Gut microbiota
IBD: Inflammatory Bowel Disease
IBS: Irritable Bowel Syndrome
IGF-1: Insulin-like growth factor 1
IL: Interleukin
IMU: Inertial Measurement Unit
IU: International Units
LDA: Linear Discriminant Analysis
LDL: Low-density lipoprotein
LEfSe: Linear discriminant analysis Effect Size
MCH: Mean Corpuscular Hemoglobin
MCHC: Mean Corpuscular Hemoglobin Concentration
MCV: Mean Corpuscular Volume
MMSE: Mini-Mental State Examination
MNA-SF: Mini-Nutritional Assessment-Short Form
MQ: General medical questionnaire
MS: Mass spectrometry
OTU: Operational Taxonomic Units
PCoA: Principal Coordinates Analysis
PERMANOVA: Permutational Multivariate Analysis of Variance
PUFA: Polyunsaturated Fatty Acids
QoL: Quality of Life
RCT: Randomized Controlled Trial
RDA: Recommended Daily Allowance
1-RM: One Repetition Maximum
SCFA: Short Chain Fatty Acids
SF-36: Short Form 36 Survey Health Questionnaire
SPPB: Short Physical Performance Battery
TEMPUS-FUGIT: Trial in Elderly with Musculoskeletal Problems due to Underlying Sarcopenia – Faeces to Unravel the Gut and Inflammation Translationally
TMT: Trail-Making-Test
TNF-α: Tumor Necrosis Factor-α
